# 3D self-supporting PANI/CNT/CC composite cathode coupled with CNF/PAM dual network gel electrolyte for high specific capacity flexible zinc-ion battery

**DOI:** 10.1186/s13065-026-01819-9

**Published:** 2026-05-02

**Authors:** Jiayi Li, Cong Wang, Kangkang Zhao, Sabeen Fatima, Xiaoxiao Zheng, Yu Sun, Muyang Li, Nan Han, Lei Han, Yafei Ning, Raffaello Papadakis, Klaus Leifer, Safia Khan, Hu Li

**Affiliations:** 1 Shandong Key Laboratory of Next-Generation Semiconductor Technology and Systems, School of Integrated Circuits, Shandong University, Jinan, 250101 China; 2 School of Energy and Power Engineering, Shandong University, Jinan, 250061 China; 3 Shenzhen Research Institute of Shandong University, Shenzhen, 518057 China; 4 Department of Forest Biomaterials and Technology, Swedish University of Agricultural Sciences, Vallvägen 9C, Uppsala, 75007 Sweden; 5 Department of Materials Science and Engineering, Uppsala University, Uppsala, 75121 Sweden; 6 Key Laboratory of Superlight Materials and Surface Technology, College of Material Science and Chemical Engineering, Harbin Engineering University, Harbin, 150001 China

**Keywords:** PANI, Double network gel electrolyte, Self-supporting structure, Flexible battery, Zinc ion battery

## Abstract

In this work, PANI/CNT/CC composite is developed as a positive electrode for flexible zinc-ion battery (ZIB) with the high specific capacity. Assembled flexible batteries are subjected to electrochemical testing. A double grid structure constructed by CNT modification of polyaniline nanofiber network on carbon cloth and CNF modification of PAM gel further improved its flexibility and bending ability. The optimized battery exhibits a high specific capacity of 210 mA hg⁻¹ at a current density of 0.5 A g⁻¹, i.e., a marked improvement in rate performance and bending stability. Following 800 charge and discharge cycles at a current density of 2 A g⁻¹, the battery demonstrates an impressive capacity retention rate of approximately 90.6% along with a notable average coulombic efficiency of 98.7%, underscoring its remarkable strength and stability. The PANI/CNT/CC|CNF/PAM|Zn battery can power the device when it is bent or even folded. Such enhanced electrochemical performance of optimized ZIB is originated from synergistic coupling between the 3D cathode and CNF/PAM dual-network hydrogel electrolyte, which simultaneously facilitates electron transport, Zn^2+^ ion diffusion, and interfacial stability.

## Highlights

1. Flexible zinc-ion battery presenting appropriate bending and cycling stability is developed.
2. 3D structured current collector is employed in the construction of a PANI composite cathode.
3. A flexible, low-impedance, high-density CNF/PAM dual-network gel electrolyte is prepared.
4. Developed ZIB exhibited long-term stability, high-capacity retention and reliable performance over extended charge-discharge cycles.

## Introduction

Surging growth of flexible, wearable, and implantable electronic devices such as flexible displays, smart fabrics, and foldable mobile phones has led to an expanding need for flexible energy storage devices with high energy and power densities [1–4]. Because of high cost of conventional lithium-ion battery, and use of toxic and flammable organic electrolytes, its safety is concerned under bending conditions [5–8], so, its further applications in wearable electronic devices are limited. There is an urgent requirement to develop flexible energy storage systems with high energy density, low cost, and environmental friendliness. Owing to advantages of low cost, non-flammability, high safety, and high ionic conductivity of aqueous electrolytes, aqueous rechargeable batteries have emerged as promising alternatives to conventional rechargeable batteries based on non-toxic organic electrolytes [7, 9, 10]. Among the aqueous multivalent metal-ion batteries, aqueous zinc-ion batteries (ZIBs) with neutral or acidic electrolyte are regarded as a new generation of inexpensive and safe energy storage devices [11] owing to better electrochemical performance, low cost and environment friendly features [9]. Battery negative electrodes are usually made directly from metallic zinc, due to the excellent compatibility of Zn with water. Zn is compatible with aqueous system when electrolyte is suitably framed and approaches to restrain dendrite formation are implemented. It reduces the water activity and aids in stabilizing the Zn^2+/^Zn redox couple by promoting ion transport and restraining side reactions. Moreover, dendrite formation in Zn is less challenging owing to lower reactivity in water. Diverse approaches e.g., surface coatings, electrolyte additives, and advanced electrode designs have given optimistic outcomes in inhibiting dendrite growth and empowering long-term cycling.

Likewise, constructing a three-dimensional structure is an effective method to inhibit the generation of dendrites, increase the surface area of the reaction, reduce the local current density, making the electric field uniformly distributed, thereby promoting a uniform deposition of metal ions [12]. By introducing the coating, the electrode/electrolyte interface can be adjusted in disguise, providing more nucleation sites and promoting the uniform deposition of zinc while mitigating side reactions. Exploitation of new gel electrolytes [13] and use of electrolyte additives [14] are expected to modulate the deposition of Zn. It has become a common approach to improve the flexibility of ZIBs by using gel polymer electrolytes (GPE) that have the combined advantages of high ionic conductivity and safety and by using three-dimensional architectures to significantly improve the performance of ZIBs [15–18].

Polyaniline (PANI) has drawn much attention owing to availability of raw materials, easy synthesis, good stability and diversified structure; however, it still offers multiple challenges when used as an anode material. The low utilization of positive electrode materials, the formation of zinc dendrites during cycling, poor electrode-electrolyte interface contact, and significant volume changes still hinders its practicality. The lower surface area/volume ratio and lower utilization rate of the positive active substance make it impossible for it to be in close contact with the collector and even leads to the detachment of the active substance, which greatly reduces the service life of a ZIB. Therefore, the exploitation of polyaniline cathode materials with high specific capacity as well as long cycle stability is an immediate concern to be addressed.

Although PANI-based cathodes and PAM-derived hydrogel electrolytes have been individually explored for aqueous zinc-ion batteries, their integrated design as a coupled electrode-electrolyte system capable of simultaneously regulating electron transport, ion transport, and mechanical stability remains insufficiently investigated. In flexible energy storage systems, electrochemical performance is strongly influenced by the compatibility between the electrode architecture and the electrolyte network. Therefore, designing a system in which both components work cooperatively to facilitate charge transport and maintain interfacial stability under mechanical deformation is essential. In this work, a 3D PANI/CNT/CC cathode is synergistically integrated with a CNF/PAM dual-network hydrogel electrolyte, forming a continuous ion-electron transport pathway. The CNT-modified carbon cloth provides a highly conductive porous scaffold for uniform PANI deposition and efficient electron transfer, while the CNF/PAM hydrogel offers improved mechanical strength, high water retention, and regulated Zn^2+^ ion transport. This coupled design enables enhanced electrochemical kinetics, improved interfacial stability, and superior flexibility in zinc-ion batteries. In this work, PANI/CC|PAM|Zn cells are constructed and modified to downplay the poor electrochemical performance of the PANI/CC composite cathode in the flexible cells as well as the poor physical properties of the gel electrolyte. Polyacrylamide (PAM) gel is an organic polymer material prepared by free radical polymerization of acrylamide (AM) monomers, and the materials are connected by covalent crosslinking and hydrogen bonds. 3D structured current collector can increase the contact area between PANI and the gel electrolyte, which not only improves the material utilization, but also increases the specific capacity of the cathode and provides more attachment sites for PANI, thereby improving the cycle performance of the battery. CNT and PANI are selected for compounding because CNT/CC composite materials have inherent hydrophilicity and high electrical conductivity, which can undoubtedly reduce the internal resistance of the battery, reduce the overpotential, and thus improve the electrochemical performance. The PAM gel electrolyte was then modified using cellulose nanofibers (CNFs), which are lightweight, tough, and biodegradable. CNF/PAM double network gel provides a continuous and highly hydrated medium, which facilitates the rapid transport of Zn²⁺ions. It helps to reduce internal resistance and improves the electrochemical performance of ZIB. The double-network hydrogel structure enhances the mechanical integrity of the electrolyte, making it more flexible. The soft and conformal nature of the hydrogel electrolyte helps suppress zinc dendrite growth by distributing the Zn²⁺ ion flux more uniformly at the electrode-electrolyte interface. Compared to liquid electrolytes, hydrogels are non-flammable and leak-resistant, enhancing the safety of the battery system. Due to its solid-like yet flexible properties, hydrogel electrolytes are ideal for the development of next-generation wearable or flexible. In this way, a dual network structure can be constructed, and CNFs improve the water retention of the hydrogel and help build a more stable transport channel. Hydrogen bonds formed because of hydroxyl groups on PAM surface introduce physical cross-linking based on the original gel system, thereby enhancing the mechanical properties of the gel and upgrading the flexibility of the gel. Our work offers a novel and optimized materials integration strategy towards flexible and stable ZIBs based upon a dual-network CNF/PAM gel electrolyte, flexible PANI/CNT/CC electrode design and system-level performance under deformation.

## Materials and methods

### Chemicals

Zinc sulfate heptahydrate (ZnSO_4_·7H_2_O), ammonium sulfate [(NH_4_)_2_SO_4_)], acrylamide (CH_2_=CHCONH), aniline (C₆H₇N), methanol (CH_3_OH), nickel nitrate (Ni(NO_3_)_2_), aluminum nitrate [Al(NO_3_)_3_], sodium dodecyl sulfate (C_12_H_25_SO_4_Na), and sodium saccharin (C₇H₄NNaO₃S) were purchased from Aladdin. N, N’-methylenebisacrylamide (MBAA) (C_7_H_10_N_2_O_2_) was provided by Ruierfeng Chemical Co., Ltd. Potassium persulfate (K₂S₂O₈), sulfuric acid (H_2_SO_4_), nickel chloride (NiCl₂), and boric acid (H₃BO₃) were purchased from Merck Co., Ltd. CNFs were provided by Huaxiang Kejie. Commercial carbon cloth (CC mainly composed of carbon, ≥ 99 wt%) was used as the current collector of the positive electrode PANI material. All chemical reagents were obtained from commercial sources and used without any further purification.

### Preparation of composite positive electrodes

CC was washed with acetone, deionized water and anhydrous ethanol respectively, and then placed in an oven to fully dry, and then cut into 2 cm × 2 cm. It was then treated under 3 V constant voltage electrolysis for 4 min. PANI was electrodeposited on the CC. In the preparation of electrolyte, 0.5 M concentrated sulfuric acid was added first, and stirred on a magnetic stirrer. Aniline monomer with a concentration of 0.1 M was slowly added and stirred to prevent the aniline monomer from agglomeration. Three-electrode system was used to deposit 15 cycles at a scanning voltage of −0.1 ~ 0.9 V and a scan rate of 20 mV/s. Electrode was then washed with deionized water and ethanol and placed in an oven at 60 ℃ to fully dry.

CNT/CC flexible composite current collector was prepared by chemical vapor deposition (CVD). CC was first subjected to the same pretreatment. Then, an electrolyte was prepared (30 g nickel sulfate, 4 g nickel chloride, 4 g boric acid, 0.1 g sodium dodecyl sulfate, and 0.4 g sodium saccharin were added to 100 mL of electrolyte). CC was held on the working electrode clamp and placed in the prepared electrolyte. Platinum electrode and saturated calomel electrode (SCE) were used as counter and reference electrodes, respectively. Constant voltage deposition was carried out at 2.05 V for 5 min, followed by cleaning and preservation in a 100 °C in oven for 30 min to obtain Ni/CC. Ni/CC was then placed in a porcelain boat, placed in a tube furnace in argon atmosphere. The temperature was raised to 800 °C at a rate of 5 °C/min, and hydrogen was introduced for half an hour to reduce the catalyst. Temperature was lowered to 700 °C and acetylene and argon were introduced as carbon source and protective gas in a volume ratio of 1:8, respectively, and deposition was carried out for 10 min to achieve the growth of CNTs. After heating, the flexible self-supporting CNT/CC composite current collector was obtained after cooling to room temperature under argon. Ni remains embedded in the substrate and may contribute to the composite’s electrical conductivity and stability. The CNT/CC composite material was used as a substrate to prepare a flexible PANI/CNT/CC composite cathode under the same cyclic voltammetry electrodeposition conditions as those for preparing PANI/CC.

### Synthesis of the GPE

AM powder and deionized water were mixed in a mass ratio of 1:10 and stirred dissolve the AM monomer. 0.1 mg/ml potassium persulfate as an initiator and 0.5 mg/ml MBAA as a crosslinker were mixed to the solution. After stirring and ultrasonication for 30 min, it was poured into a special mold and placed in a vacuum oven and let it stand at 60 °C for 4 h, and the cross-linked CNF/PAM double network gel. Aqueous gel electrolyte was prepared by taking 1 M ZnSO_4_ solution and adding ammonium sulfate buffer to adjust the pH at 4. Cross-linked PAM hydrogel was finally soaked in electrolyte for 2 h to ensure its good conductivity and low interfacial impedance. CNFs were mixed with deionized water at a mass ratio of 1:100 and stirred thoroughly. CNF/PAM double network gel was poured into 1 M ZnSO_4_ and soaked in electrolyte with a pH of 4 using (NH_3_)_2_SO_4_ buffer for 2 h. During the cross-linking process, AM monomers undergo free radical polymerization and get adsorbed on CNF by connecting with many hydroxyl and carboxyl bonds on the CNF surface, finally obtaining a CNF/PAM double network gel electrolyte.

**Fig. 1:**
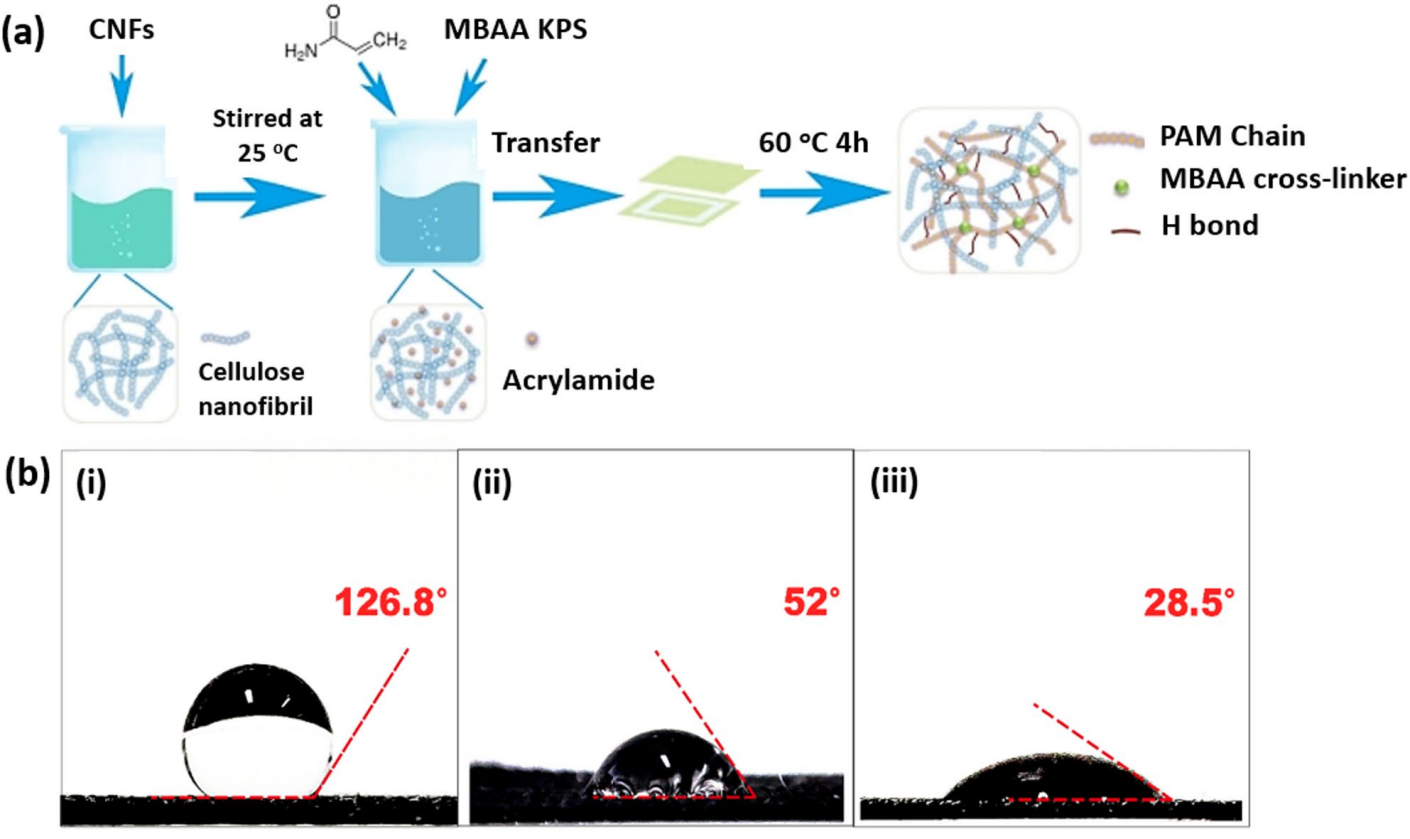
Schematic diagram of synthesis of CNF/PAM hydrogel dual network structure (**a**). Measurement of water surface contact angle at CC; before treatment (i), for 2 min (ii) and for 4 min (iii) (**b**)

The surface of CNFs contains hydroxyl (-OH) and carboxyl (-COOH) groups, which provide active sites for interaction with PAM chains. AM monomers polymerize and crosslink through MBAA, forming a second polymer network interpenetrating the CNF structure. PAM chains partially adsorb onto CNFs through hydrogen bonding and possibly ionic interactions between amide groups and carboxyl groups on CNFs. This results in strong interfacial adhesion between the two networks. This synergistic structure significantly enhances mechanical strength, ionic conductivity, and structural integrity of the gel electrolyte. The mechanism of formation of GPE is presented in Fig. 1a and Eqs. (1–4).

Potassium persulfate decomposes upon heating to produce sulfate radicals:

1 $${\mathrm{K}}_{{\mathrm{2}}} {\mathrm{S}}_{{\mathrm{2}}} {\mathrm{O}}_{{{\mathrm{8}}}} \to {\mathrm{2K}}^{ + } + {\mathrm{2SO}}_{{\mathrm{4}}} ^{{ - }}$$

The sulfate radicals initiate free radical polymerization of acrylamide (AM):

2 $$ {\mathrm{SO}}_{{\mathrm{4}}} ^{{ \cdot {-}}} + \,{\mathrm{CH}}_{{\mathrm{2}}} = {\mathrm{CHCONH}}_{{\mathrm{2}}} \, \to {\mathrm{CH}}_{{\mathrm{2}}} ^{ \cdot } {\mathrm{CHCONH}}_{{\mathrm{2}}} $$

The radical on the acrylamide monomer reacts with more acrylamide monomers to grow the polymer chain:

3 $$ {\mathrm{CH}}_{{\mathrm{2}}} \cdot {\mathrm{CHCONH}}_{{\mathrm{2}}} + {\mathrm{n}}({\mathrm{CH}}_{{\mathrm{2}}} = {\mathrm{CHCONH}}_{{\mathrm{2}}} ) \to [ - {\mathrm{CH}}_{{\mathrm{2}}} {\mathrm{CHCONH}}_{{\mathrm{2}}} ^{ - } ]_{{\mathrm{n}}} ^{ \cdot }$$

MBAA has two double bonds and acts as a crosslinker, linking PAM chains:

4 $$ \begin{aligned} {\mathrm{PAM}}^{{ \cdot - }} + {\mathrm{CH}}_{{\mathrm{2}}} = & {\mathrm{CH}} - {\mathrm{CONH}} - {\mathrm{CH}}_{{\mathrm{2}}} - {\mathrm{NHCO}} - {\mathrm{CH}} \\ = & {\mathrm{CH}}_{{\mathrm{2}}} \to ~~{\mathrm{Cross}} - {\mathrm{linked}}{\mathrm{PAM}}{\mathrm{network}}~ \\ \end{aligned} $$

The strong hydrogen bonds between the PAM amide (-CONH₂) and CNF hydroxyl (-OH), contribute to physical cross-linking. These interactions form the double-network structure without needing full covalent bonding.

### Battery assembly and testing

After the zinc foil is fully flattened, it was cut into a size of 1 cm × 2 cm, cleaned with alcohol, dried, and taken out for use. The flexible device is assembled according to the sandwich structure. First, the zinc foil is placed on the inner surface of the aluminum foil ziplock bag. Then, the gel electrolyte is clamped with tweezers to align with zinc foil. Composite cathode with the active material side facing down is laid flat on the electrolyte. Finally, after a pole ear is led out from each positive and negative electrode, the battery is sealed with a sealing machine. A flexible PANI-zinc ion battery is obtained. The samples were characterized by a range of analytical techniques, including field emission scanning electron microscopy (FE-SEM, HITACHI S-4800), transmission electron microscopy (TEM, JEM-2100), X-ray photoelectron spectroscopy (XPS, Escalab 250Xi), Raman spectroscopy (Raman, InVia-Reflex), Fourier transform infrared spectroscopy (FT-IR, Nicolet 8700 FTIR), a contact angle meter (Dataphysics OCA40) and X-ray diffraction (XRD, DX-2700). Cyclic voltammetry (CV), constant current galvanic charge and discharge (GCD), and electrochemical impedance spectroscopy (EIS) of a single electrode were studied on an electrochemical workstation. Subsequently, linear sweep voltammetry (LSV), constant current GCD, and EIS were performed on the gel electrolyte. Finally, the performance of the full battery system was evaluated.

## Results and discussion

### Physical characterization

The application of constant voltage electrolysis at 3 V to commercial carbon cloth results in a treatment time of between one and six minutes. In Fig. 1b, 3 V constant voltage electrolysis has been demonstrated to markedly enhance hydrophilicity within the initial 4 min, with a notable reduction in the water surface contact angle as the electrolysis time increases. Following a period of more than 4 min, the water surface contact angle continued to decrease.

**Fig. 2:**
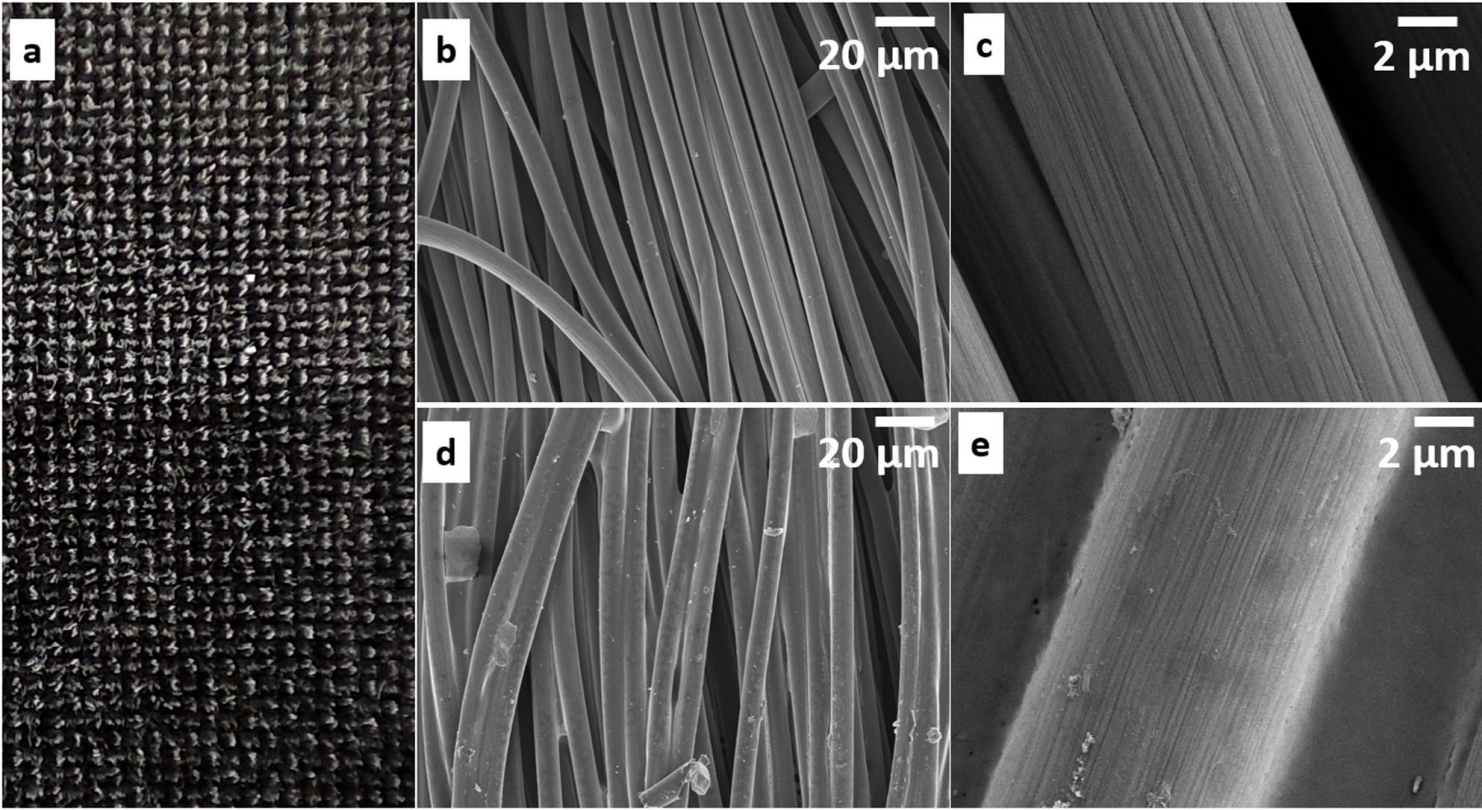
Optical photos of CC before and after processing (**a**). SEM images of CC before (**b–c**) and after processing (**d**–**e**)

Electron micrographs showed that the surface of the carbon fiber undergoes a transformation following electrochemical treatment as illustrated in Fig. 2. The surface becomes uneven, exhibiting an increased number of cracks and the formation of surface oxide layers. This alters the surface area to volume ratio of the carbon cloth, creating additional reaction sites for chemical reactions and enhancing the hydrophilicity and electrochemical performance of the material. Furthermore, the fibers of the carbon cloth are broken, which is tantamount to a reduction in the surface area of the cloth, resulting in a decline in conductivity.

**Fig. 3:**
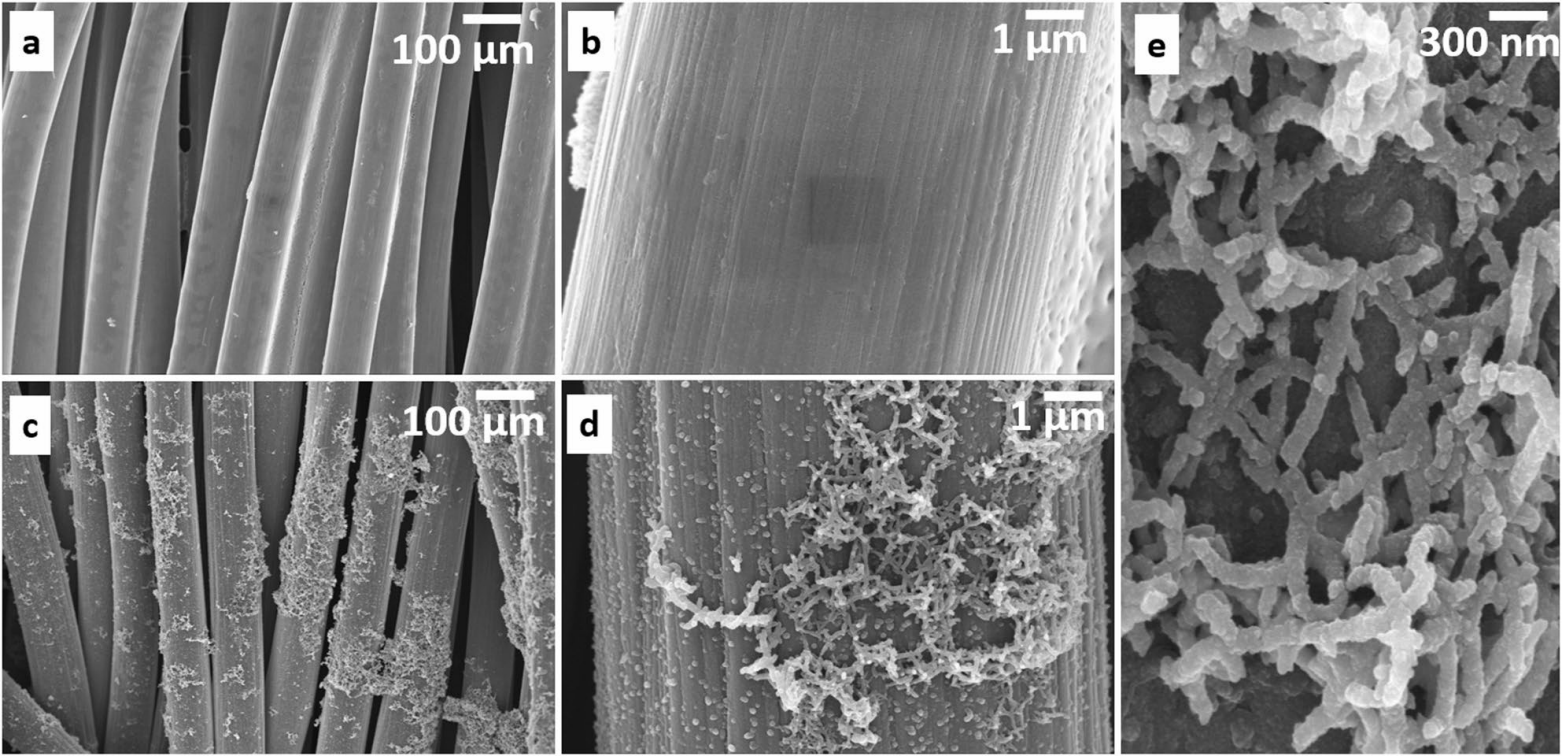
SEM images before (**a–b**) and after deposition (**c–d**). Morphology of PANI after deposition under high-power microscopy (**e**)

The aggregated PANI will continue to grow and connect to each other, forming a coral bone-like grid structure with a diameter of approximately 80 nm as presented in Fig. 3. The formation of such a nanostructure can result in an increase in the specific surface area of PANI, which in turn can lead to improvements in its electrochemical performance.

**Fig. 4:**
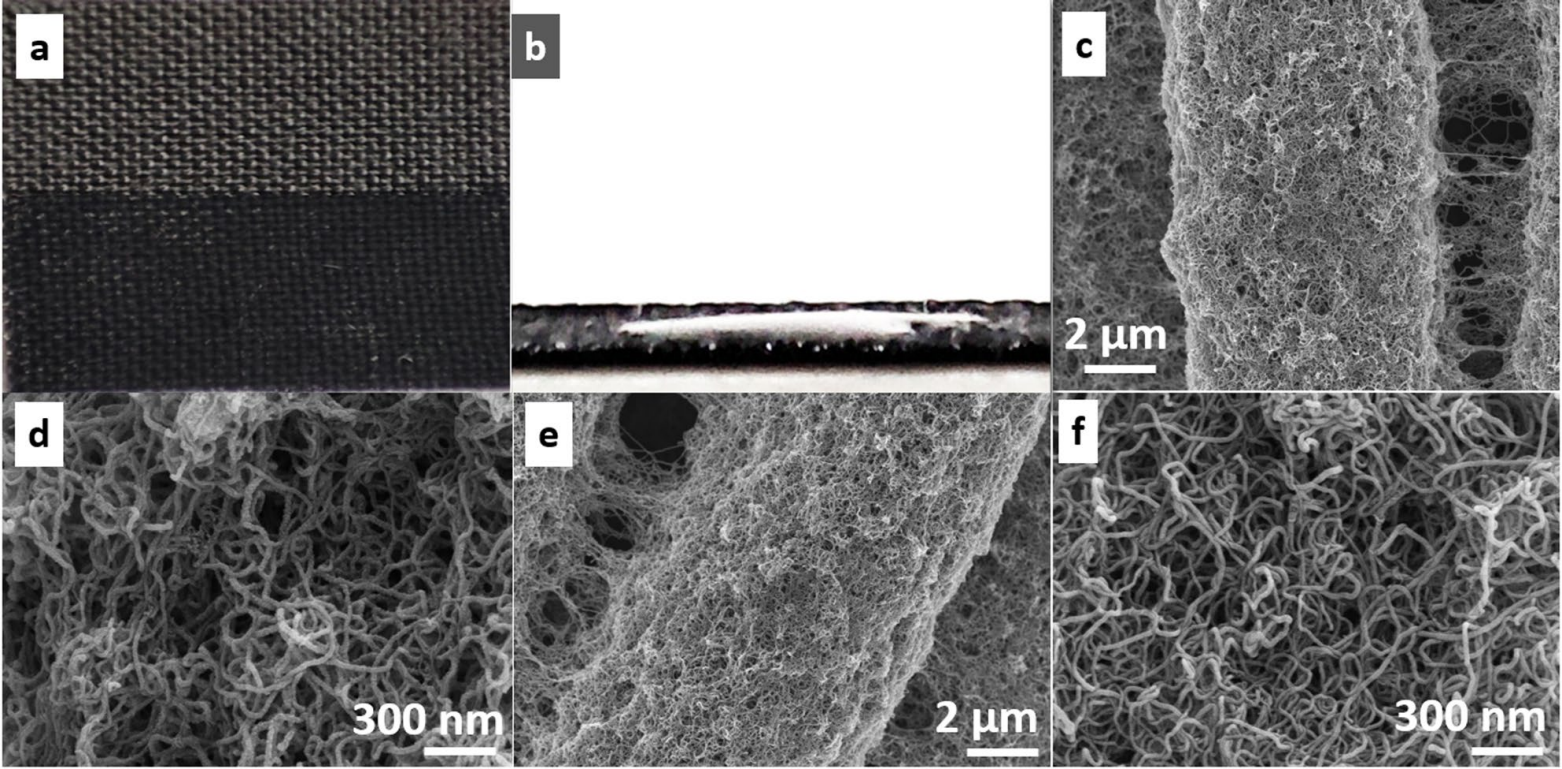
Comparison of optical photos between CC (upper) and CNT/CC (lower) (**a**) Water surface contact angle test of CNT/CC (**b**) SEM characterization of CNT/CC (**c–d**) SEM micrographs of PANI/CNT/CC (**e–f**)

Figure 4a shows the optical photo comparison between CC and CNT/CC, the contact angle, and the SEM characterization of CNT/CC and PANI/CNT/CC. It can be seen from Fig. 4b that the CNT/CC composite cathode significantly improves the hydrophilicity of the carbon cloth, and the water contact angle is close to 0°. Following testing, it was found that the water absorption rate of the CNF/PAM double network gel could reach approximately 1265%, representing a 55% increase in comparison to the absorption rate of the PAM gel. This is because the CNF network introduces a considerable number of hydrophilic groups. SEM micrographs presented in Fig. 4c and d show that the interconnected CNT highly conductive network with a diameter of about 30 nm is evenly distributed on the CC surface, forming a surface regular and interconnected conductive network, successfully establishing a flexible and conductive skeleton that can be used to uniformly deposit PANI. The surface morphology of CNT did not change significantly after the deposition of PANI Fig. 4e and f. Like PANI/CC, a uniform film-like structure appeared on the surface of the CNT after the deposition, which made the previously rough surface smooth, and there was no agglomeration as seen on the carbon cloth. It indicates that three-dimensional current collector with high conductivity and a high ratio of surface area to volume is conducive to the uniform deposition of PANI. Its structure can increase the specific surface area, reduce the local current density on the PANI surface during battery charge and discharge, make the electric field uniform in the cathode area, and absorb PANI shed due to volume expansion and contraction during the cycle, thereby improving the electrochemical performance of the flexible cathode.

**Fig. 5:**
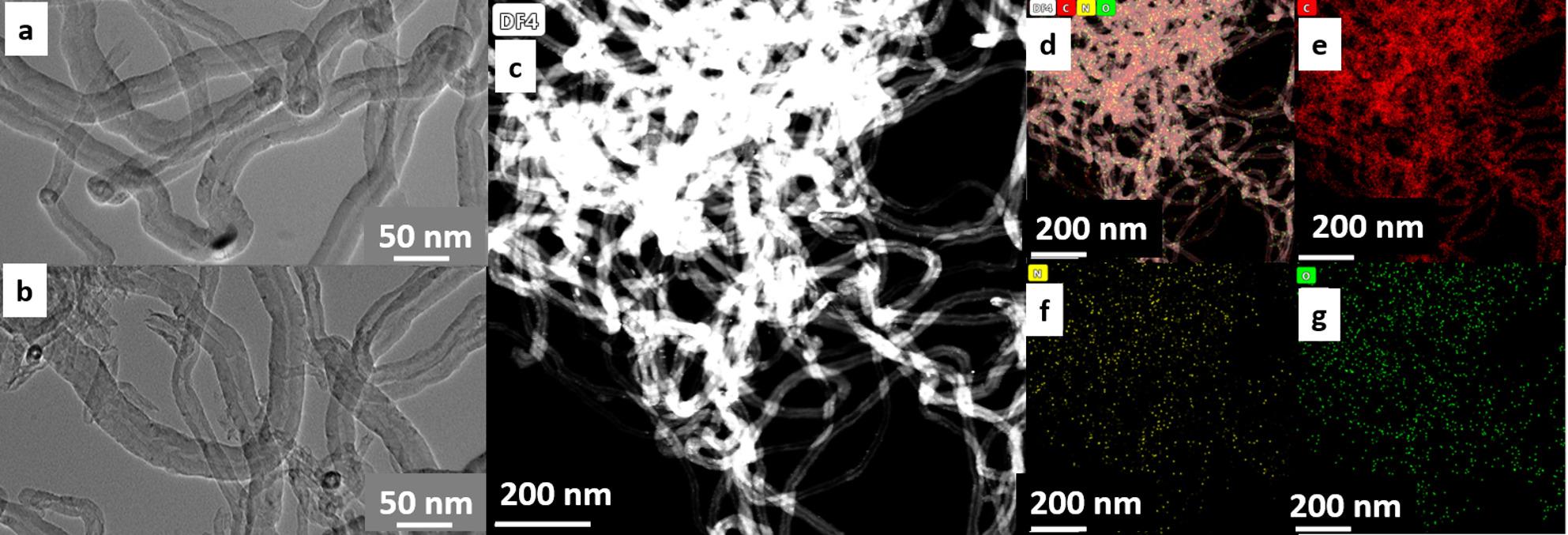
TEM images of CNT (**a**) and PANI/CNT samples (**b**). TEM selected-area elemental mapping images of PANI/CNT (**c–g**)

The corresponding inference can be drawn from TEM images of the CNT/CC composite current collector and the PANI/CNT/CC flexible cathode and the elemental distribution diagram of the flexible cathode (Fig. 5). After the deposition (Fig. 5b), a PANI film structure is formed on the surface of the CNTs in comparison with the situation before the deposition (Fig. 5a). PANI/CNT has a smaller diameter of about 30 nm compared to the coral-like lattice structure with a diameter of about 90 nm formed by PANI on CC. This further evidence that the design of the composite current collector can not only form a 3D highly conductive PANI/CNT network but also increase the specific surface area of the active material to obtain a flexible positive electrode with better electrochemical performance. Further confirming the successful synthesis of PANI/CNT/CC flexible electrodes, the selected area element mapping (Fig. 5c–g) shows that the C, N and O elements are evenly distributed.

**Fig. 6:**
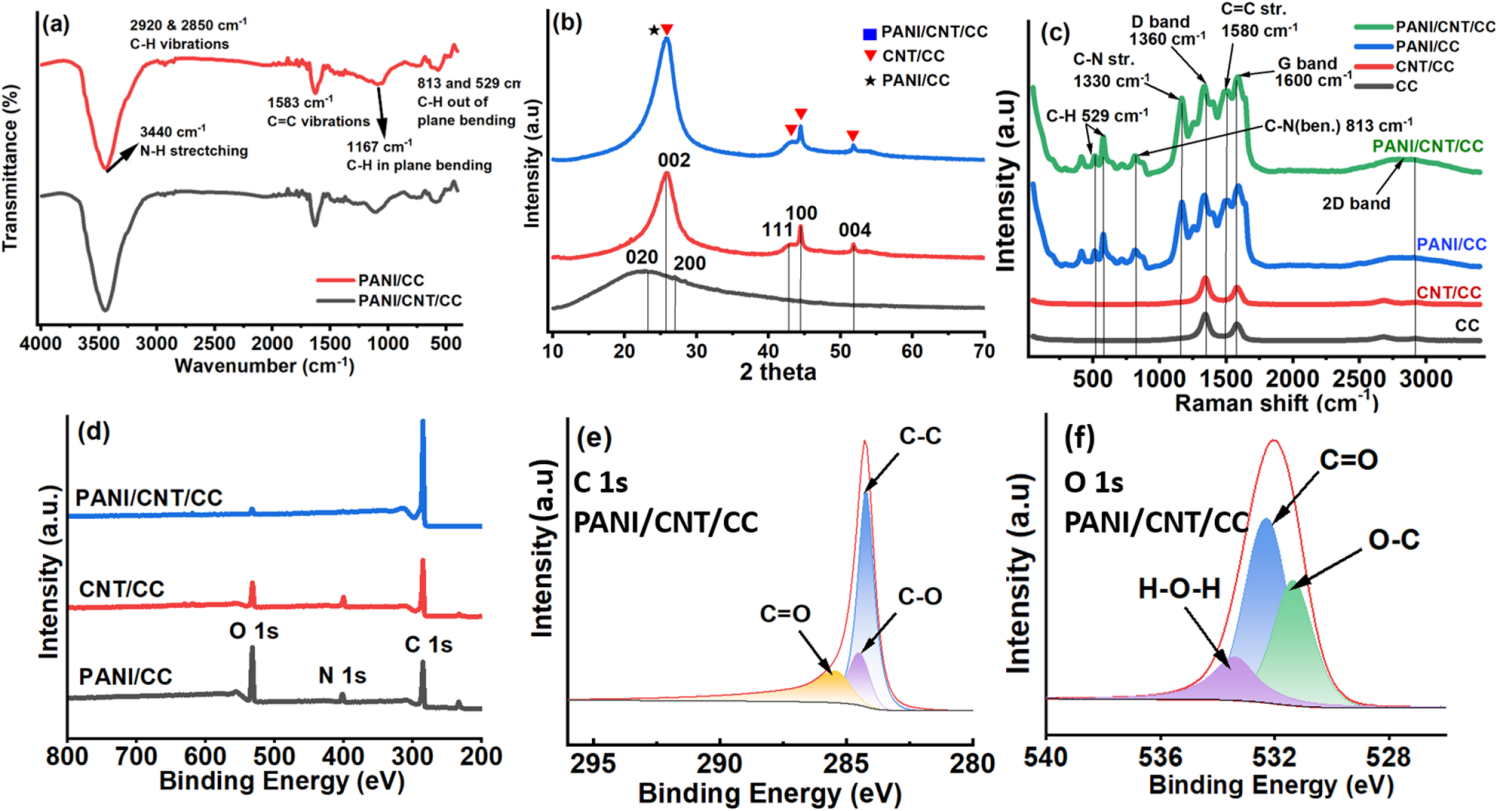
FTIR spectra of PANI/CC and PANI/CNT/CC (**a**). XRD patterns of PANI/CC, CNT/CC and PANI/CNT/CC (**b**). Raman Spectra of all composite cathodes (**c**). XPS survey spectra of PANI/CC, CNT/CC and PANI/CNT/CC (**d**) deconvoluted spectra of C 1 s (**e**) O 1 s (**f**) of PANI/CNT/CC composite

FTIR analysis of CC is shown in Fig. 6a. CC exhibited an absorption peak of -OH at a wavelength of approximately 3450 cm^− 1^ following constant voltage electrolysis. Concurrently, a peak indicative of a -C-O- stretching vibration was observed at 1100 cm^− 1^ [19], while a -C = O characteristic peak manifested at approximately 1650 cm^− 1^ [20]. The results demonstrate that the surface of CC undergoes oxidation following electrochemical treatment, resulting in the formation of functional hydrophilic oxygen-containing groups. This process significantly enhances the hydrophilic properties of the material. Appearance of absorption peaks at wavelengths of approximately 1568 cm^− 1^ and 1489 cm^− 1^ indicates the stretching vibration of the C = C bond in the quinone ring and benzene ring of PANI [21]. The absorption peaks at approximately 1131 cm^− 1^ and 803 cm^− 1^ are attributed to the in-plane bending vibration of C-H in the benzene ring and the out-of-plane bending vibration of C-H in 1,4-disubstituted benzene [22]. The absorption peak at 3445 cm^− 1^ is attributed to the stretching vibration of N-H [23]. The aforementioned absorption peaks are indicative of doped PANI functional groups, thereby substantiating the successful preparation of PANI/CNT/CC [24].

Furthermore, the appearance of crystalline peak in XRD patterns in Fig. 6b at ~22° corresponds to (020) crystal plane diffraction peak of the emeraldine salt form of PANI. It corroborates the successful synthesis of PANI on CC in the form of doped conductive emeraldine salt [24, 25]. Diffraction peak in PANI/CNT/CC at 26° owes to the (002) plane of graphitic carbon i.e., CNTs or CC [26]. Peak appeared at 43° and 52° arise from nickel collector i.e., Ni (111) and Ni (200) planes [27, 28]. Appeared peaks confer the semi-crystalline structure of PANI and the presence of carbon phases of CNT/CC. PANI/CNT/CC displayed more intense peaks at 22°, 26°, and 43° than PANI/CC and CNT/CC. It suggested that CNTs endorse better nucleation sites leading to crystallinity.

From the Raman spectra in Fig. 6c, both the CC and CNT/CC spectra have only two peaks characteristic of the core of the two carbon materials, namely the D peak at ~1360 cm^− 1^ and the G peak at ~1600 cm^− 1^. The ratio of the G peak to the D peak (1/R = I_D_/I_G_) can be used to characterize the degree of graphitization of carbon materials. A larger R value indicates a greater degree of disorder in the material. The quality and crystallinity of CNTs can be roughly measured by the R value. The prepared CNT/CC composite current collector exhibits a relatively low R value (0.79), indicating a higher degree of graphitization and better structural order due to catalytic growth of CNTs. After electrodeposition, both PANI/CC and PANI/CNT/CC show slightly increased ID/IG values, suggesting the introduction of structural disorder due to the incorporation of PANI. However, the PANI/CNT/CC composite retains a relatively low ID/IG compared to PANI/CC, signifying better graphitic quality owing to the interaction between PANI and CNT. Peak at 1330 cm^− 1^ may be attributed to the C-N^+^ stretching vibration, suggesting that the PANI present within the composite cathode is in a doped state. The peak at 1583 cm^− 1^ is identified as the quinone ring C = C stretching peak. It is possible that these two peaks may overlap with the D and G peaks of the current collector, whether CC or CNT/CC. Furthermore, new PANI characteristic peaks appeared at 1628, 1583, 1512, 1488, 1395, 1245, 1167, 813, 589, and 529 cm^− 1^, corresponding to the C-C stretching vibration of the benzene ring, the C = C stretching vibration of the quinone ring, the in-plane bending vibration of N-H, the C = N stretching vibration of the quinone ring, the stretching vibration of C-N of the benzene ring, the C-H bending deformation of the quinone ring, the in-plane bending vibration of C-H in the semiquinone ring, the deformation vibration of the benzene ring, and the out-of-plane C-H deformation of the quinone ring [103]. The 2D band, typically around ~2700 cm⁻¹, is weak in the spectra, which is expected due to the multi-walled nature of the CNTs and the amorphous carbon structure in CC. In single-layer graphene, the 2D peak is sharp and intense, but herein the broad and low-intensity 2D region confirms the presence of multi-walled CNTs and/or low crystallinity in carbon materials. In contrast to the characteristic peaks of PANI/CC, the positions of these peaks in PANI/CNT/CC have undergone a certain degree of shift, which signifies that PANI can form a specific interaction with the CNT/CC current collector. This interaction is presumed to be the π-π attraction between CNT and PANI, which is anticipated to enhance the overall performance of the composite positive electrode [29–31].

The XPS survey spectra is presented in Fig. 6d. In both PANI/CC and PANI/CNT/CC, only C 1 s, O 1 s, and N 1 s signals are observed, indicating that the prepared PANI is of high purity [32–34]. The C 1 s and O 1 s of PANI/CNT/CC were subjected to further fitting, and C 1 s peak was found to be consistent with five components as expressed in Fig. 6e, i.e., identified as C = C or C–C (284.8 eV), C-N (284.7 eV), C-O-C (285.6 eV), O = C-O (288 eV) and π-π* vibration peak (290.6 eV) [35–38]. The absence of the N 1 s peak in the XPS analysis of PANI/CNT/CC could be due to surface coverage effects or low nitrogen content that falls below the detection threshold. The characteristic peaks of the sample were observed to shift when compared to the control sample, which may be attributed to the formation of conjugation and the augmentation of hydrogen bonds between PANI and CNT. The formation of conjugation can be accounted for by the electrostatic interaction of the carboxyl groups of the CNTs with the main chain of the PANI, as well as the π-π stacking of the PANI and CNTs [39]. The presence of hydrogen bonds can be attributed to the presence of oxygen and nitrogen functional groups in the composite. The π-π* oscillation satellite peak is a distinctive feature of aromatic or conjugated systems, and its emergence indicates an enhancement in the conjugation within the composite [40]. The O 1 s spectrum shown by Fig. 6f can be fitted into three peaks at 533.8, 532.3 and 531.2 eV [113,115]. The experimental results collectively demonstrate the successful preparation of high-quality PANI on the CNT/CC surface, as well as the interaction between PANI and CNT.

**Fig. 7:**
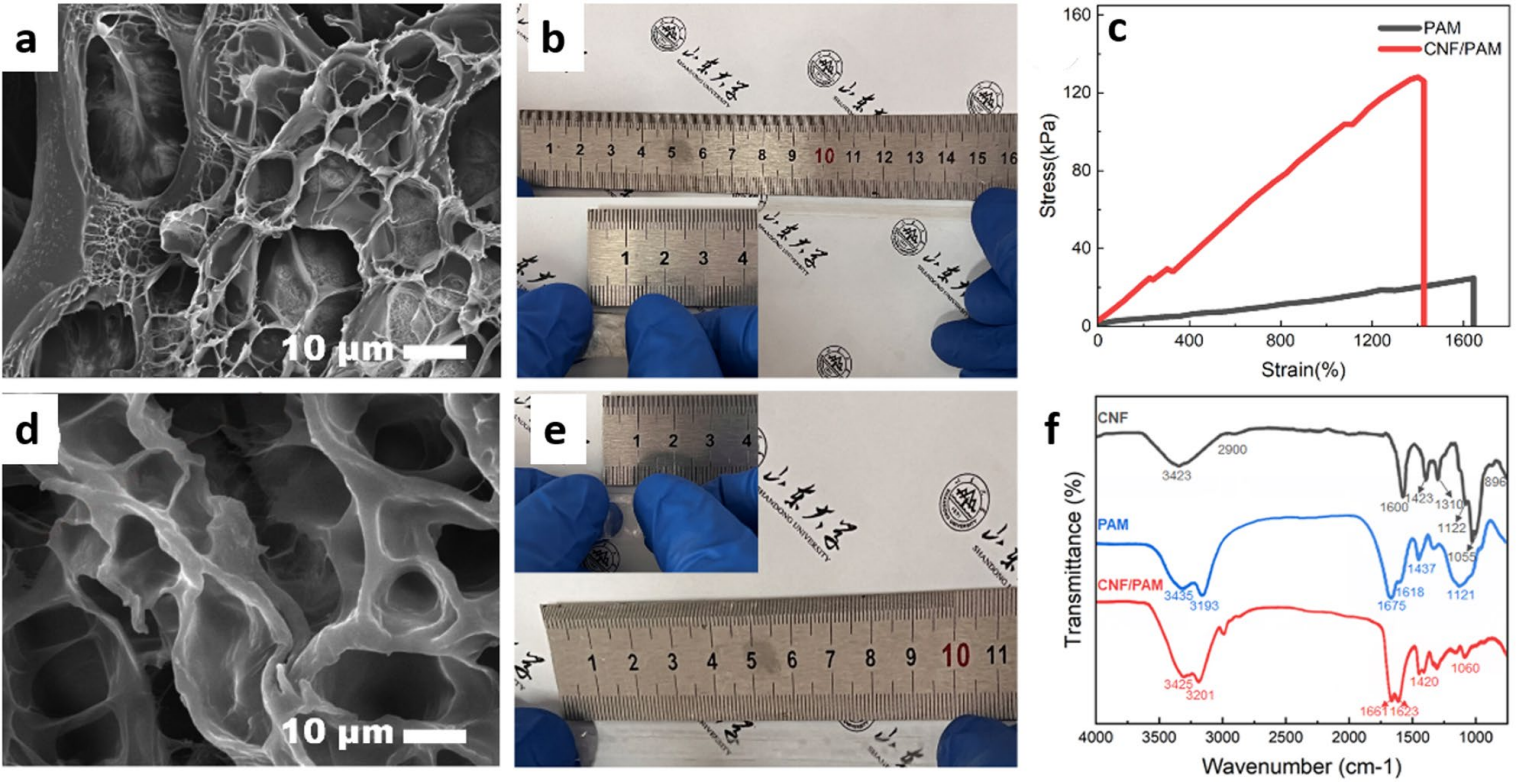
SEM images of PAM (**a**) CNF/PAM and (**b**) demonstration of tensile properties of PAM (**c**) CNF/PAM (**d**) stress-strain curve (**e**) and FT-IR spectrum (**f**)

The freeze-dried PAM film exhibits a microporous structure with a diameter of approximately 5 to 10 µm as illustrated in Fig. 7a. In contrast, the pore diameter of the CNF/PAM composite gel is observed to be between 5 and 30 μm as in Fig. 7b, which is significantly larger than that of PAM. In the CNF/PAM double network gel electrolyte, the CNF network serves as a stable three-dimensional framework, with PAM anchored on the surface of the CNF network. The PAM chain and the CNF chain are interwind to form a double network structure. The porous structure facilitates rapid carrier transmission, enabling the electrolyte to traverse the polymer skeleton with minimal delay, thereby ensuring the high ionic conductivity of the CNF/PAM double network gel electrolyte. Concurrently, the –NH of PAM will form a multitude of hydrogen bonds with the –OH of CNF. The formation of these hydrogen bonds serves to dissipate energy when the material is subjected to stress, thereby greatly improving the stability of the dual network structure. When subjected to tensile forces, the curled CNF macromolecular chains within the PAM skeleton can dissipate energy through a process of straightening. These two mechanisms of energy dissipation are reversible during the expansion and contraction of the hydrogel. It can therefore be concluded that the PAM skeleton will not break during the continuous expansion and contraction of the hydrogel, thus ensuring the stability of the hydrogel structure. Figure 7c and d show the mechanical strength of PAM and CNF/PAM, respectively and the stress-strain curves are depicted by Fig. 7e. These images and curves show that the CNF/PAM hydrogel can be easily stretched to 1000% without any cracks or breakage. The mechanical strength of pure PAM is relatively low, at 36.5 kPa. However, following the addition of CNF, the strength can be increased to 137 kPa, representing a fourfold increase over pure PAM. This demonstrates that interweaving and entanglement effect between the cellulose skeleton and PAM chains, along with the formation of hydrogen bonds, can markedly enhance the mechanical stability of the electrolyte, thus aligning better with the requirements of flexible ZIBs.

Further structure of CNF/PAM gel was confirmed by FTIR spectroscopy, as illustrated in Fig. 7f. CNF exhibits a pronounced absorption band at 3425 cm^−1^, which can be attributed to the -OH stretching vibration [41, 42]. The absorption peak observed at 2900 cm^−1^ is attributed to the stretching frequency of C-H, while the absorption peak at 1610 cm^−1^ serves to confirm the presence of COO^-^ groups. The broad band observed at approximately 3435 cm^−1^ in the PAM gel spectrum is indicative of N-H stretching, which provides evidence for the presence of NH_2_ groups. The absorption peaks at 1675 and 1616 cm^−1^ are indicative of the presence of two amide groups. The C = O stretching vibration (amide I) and N-H bending (amide II) are also observed [43–45]. In addition, the absorption peaks near 1423 and 1122 cm^−1^ are caused by CH_2_ shearing and twisting, and the peak at 896 cm^−1^ is the rocking vibration peak of N-H. In contrast, the infrared spectrum of CNF/PAM showed absorption peaks at 3425 and 3201 cm^−1^, which is the result of the superposition of the N-H stretching peaks of PAM and the -OH stretching peaks of CNF [44, 46]. Concurrently, the typical spectral bands of the amide group of PAM and the COO^-^ group of CNF overlap, resulting in the formation of absorption peaks at 1661 and 1623 cm^−1^ [47]. CNF/PAM does not show a new absorption peak, indicating that PAM and CNF are connected through physical entanglement and hydrogen bonds. The absorption peak in CNF/PAM is like CNF and PAM, proving that CNF and PAM are successfully entangled and cross-linked.

### Interface stability of CNF/PAM double network gel electrolyte and Zn anode

**Fig. 8:**
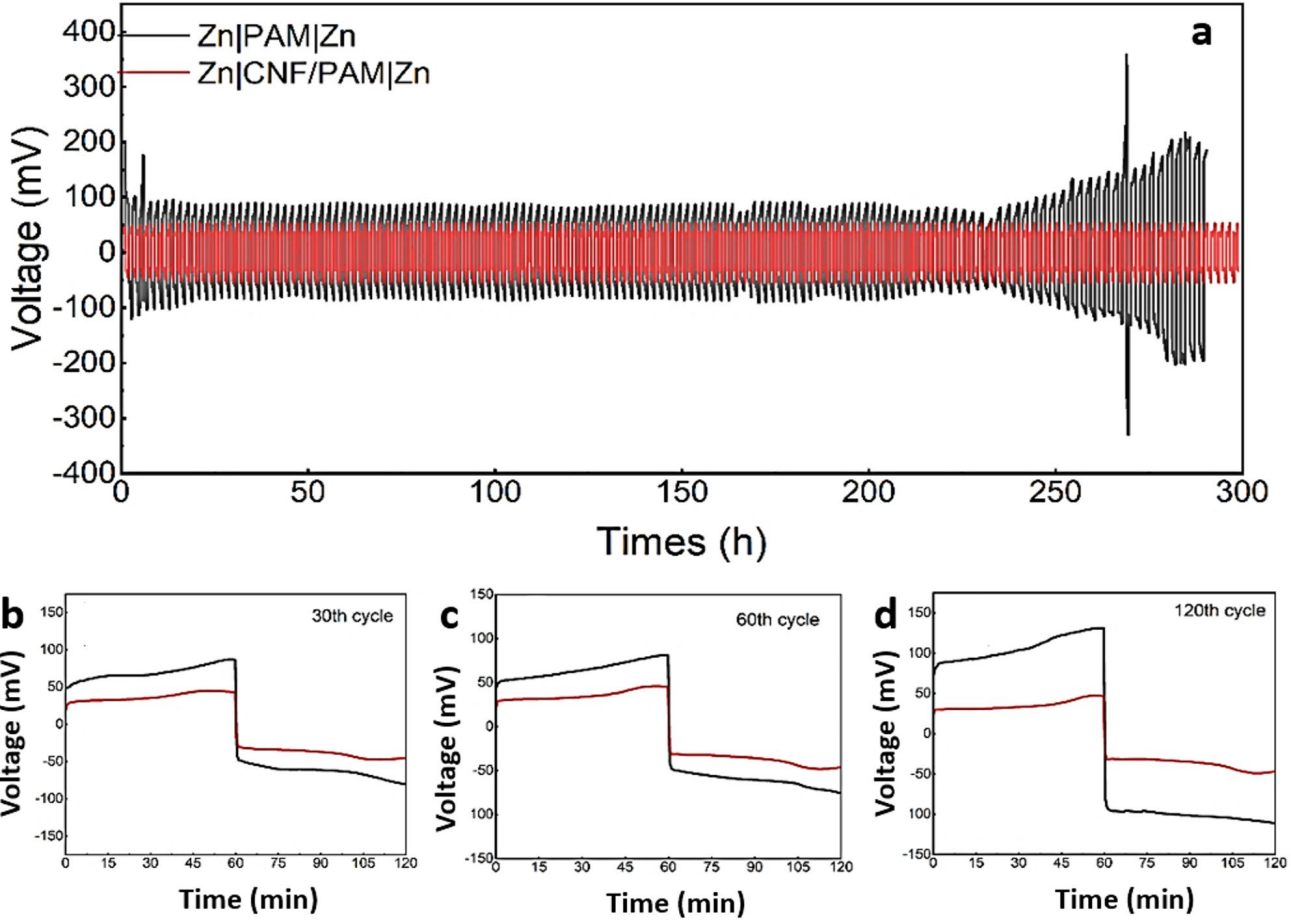
Constant current charging and discharging experiment of zinc//zinc symmetric battery enlarged view of voltage distribution (**a**) comparison of constant current charging and discharging curves in CNF/PAM based electrolyte and PAM based electrolyte (**b–d**)

To assess the interfacial stability between the zinc electrode and electrolyte, a Zn|CNF/PAM|Zn symmetric cell was tested under a constant current density of 0.5 mA cm⁻². Under constant current charge and discharge conditions of 0.5 mA cm^−2^, the Zn|CNF/PAM|Zn symmetric battery was assembled using CNF/PAM double network gel electrolyte as ion transport medium. As illustrated in Fig. 8a, the voltage of the Zn|PAM|Zn symmetric battery, assembled with PAM hydrogel electrolyte, demonstrated a relatively stable profile within the initial 215 h of the constant current charge and discharge experiment, conducted at a current density of 0.5 mA cm^−2^. Upon exceeding the 215 h cycle time, the overpotential exhibited a notable increase, indicating a concomitant decline in battery stability. The Zn|PAM|Zn cell exhibited stable cycling for the first ~215 h, after which a sudden increase in overpotential suggested growing interfacial resistance likely due to dendritic Zn growth and dead zinc formation. In contrast, the Zn|CNF/PAM|Zn cell maintained a low and stable overpotential (~45 mV) for over 300 h, clearly demonstrating superior interfacial stability and suppressed dendrite formation. Voltage distribution magnification diagram illustrates that the symmetrical battery, comprising a CNF/PAM dual network gel electrolyte, can maintain stability throughout a 300-h cycle without exhibiting signs of a battery short circuit or a rapid increase in overpotential. This evidence conclusively demonstrates that, in comparison with the PAM gel, the zinc-negative electrode and CNF/PAM double network gel electrolyte exhibit a reduction in interfacial impedance and an enhancement in interfacial stability. The polarization voltage analyzed from Fig. 8b, c and d of the symmetrical battery assembled with CNF/PAM hydrogel electrolyte can be stabilized at approximately 45 mV. In contrast, the polarization voltage of the symmetrical battery assembled with PAM hydrogel electrolyte is larger and exhibits greater instability. Inclusion of CNFs introduces abundant carboxylic functional groups that interact with Zn²⁺ ions, promoting uniform ion distribution and deposition. These functional groups also act as coordination sites, guiding Zn²⁺ migration and mitigating localized ion accumulation that often triggers dendrite nucleation. Physically robust CNF/PAM matrix can withstand volume fluctuations and mechanically suppress dendrite penetration.

The de-plating process of zinc ions on the surface of a zinc electrode can be regulated when a CNF/PAM double network hydrogel electrolyte is employed. The carboxylic acid groups can interact with the zinc ions, thereby constraining the deposition of zinc ions on the surface of the zinc negative electrode. The carboxylic acid groups function as constraint chains, facilitating the binding of ions and guiding ion migration [48]. The double grid structure of CNF/PAM is conducive to regulating the deposition process of Zn^2+^ in the negative electrode area, which has the effect of reducing the local concentration of zinc ions at the negative electrode protrusion. It leads to slowing down of growth of zinc dendrites. Furthermore, the superior physical properties of the CNF/PAM gel enable it to exert a degree of inhibition on the growth of zinc dendrites.

### Electrochemical performance of flexible PANI/CNT/CC|CNF/PAM|Zn battery

**Fig. 9:**
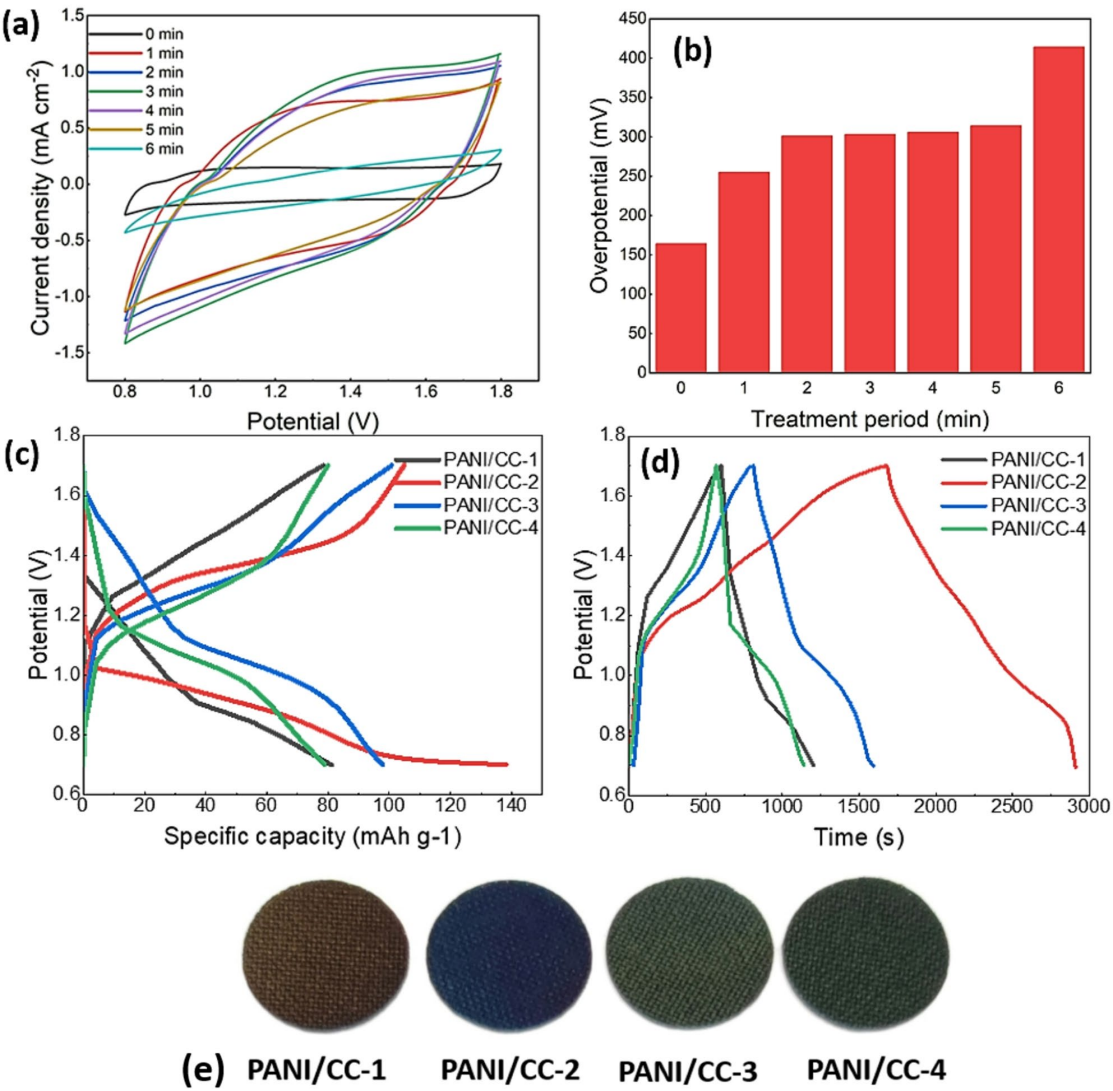
CV of CC under different processing times at 50 mV s^− 1^ (**a**) Change in overpotential (**b**) Specific capacity - voltage curve of the first charge and discharge and (**c**) Voltage time curve of four samples (**d**)

 Figure 9a shows that the current density reaches a maximum value at the three-minute mark, after which it gradually declines. The turning point is reached at the six-minute mark, resulting in a significant decline in the electrochemical performance of the carbon cloth. This may be attributed to the fracturing of the fibers within the carbon cloth because of the prolonged treatment period, which impedes the transfer of electrons. The optimal treatment time is determined to be four minutes. Table 1 demonstrates that CC was doped with sulfur while undergoing oxidation, which also resulted in a notable increase in the specific capacity of CC. Figure 9b indicates the variation in overpotential with increase in time period of scans. Initial low overpotential at 0 min indicates an instant wetting and unhindered access of ions to the highly conductive bare CNT/CC surface. With increasing polymerization, bare CNT/CC sites are obstructed hence overpotential increased at 1 min. Furthermore, a relevantly constant overpotential observed from 2 to 5 min reflects a balance between conductivity and stability. At 6-min interval, the overpotential inclined significantly owing to overgrowth of thick PANI layer, slow diffusion elevated concentration polarization and reduced effective surface area. It indicates an optimal polymerization window between 2 and 5 min where redox activity and interfacial stability are dominant without incurring transport resistance. An optimal treatment duration of four minutes laid a balance by maximizing current density while minimizing fiber damage and decrease overpotential thereby ensuring stable electrochemical behavior.

**Table 1:** Elemental content of CC before and after treatment

Sample (CC)	C	O	N	S
Before treatment（wt％）	92.67	5.01	2.32	0
After treatment（wt％）	70.86	21.08	3.55	4.5

Figure 9c refers to a galvanostatic charge–discharge profile of PANI/CC composites. It reveals an increase in the concentration of sulfuric acid accompanied by an initial rise in the specific capacity of the PANI positive electrode, followed by a subsequent decline. The lower specific capacity of PANI/CC-1 can be attributed to the low active material loading and insufficient oxidation. The oxidation peak intensity and area under the curve increased with sulfuric acid concentration up to 0.5 M (PANI/CC-2), beyond which over-oxidation and inefficient material utilization caused reduced performance (Fig. 9d). H₂SO₄ acts as a dopant, facilitating the protonation of the PANI chains, which enhances the conductivity by increasing the number of free charge carriers within the polymer. As the concentration of H₂SO₄ increases, the doping efficiency improves, leading to better conductivity of the PANI. Regarding the increased conductivity observed, this is indeed partly attributable to the optimal coating of PANI. In this way, despite the relatively high conductivity of PANI/CC-3 and PANI/CC-4, the utilization rate of cathode material is comparatively low in comparison to PANI/CC-2. This results in a reduction in the specific capacity of the former. PANI/CC-2 exhibits highest specific capacity, reaching ~140 mA hg^− 1^ owing to optimal loading of PANI. In the subsequent experimental design, this condition was employed in the preparation of the PANI cathode. The sulfuric acid concentration in the electrolyte was set to 0.25 M, 0.5 M, 0.75 M and 1 M, respectively during the electrodeposition process to obtain four groups of samples. The samples were designated PANI/CC-1, PANI/CC-2, PANI/CC-3 and PANI/CC-4. The four samples in Fig. 9e exhibited a range of colors, from yellow brown to dark green, indicating that as the concentration of concentrated sulfuric acid increased, the degree of PANI oxidation also increased, and the amount of deposition increased in proportion. As indicated in Table 2, an increase in the concentration of H₂SO₄ is accompanied by an enhancement in the conductivity of PANI. Upon reaching a concentration of 0.5 M, the conductivity of the electrode remains largely unaltered. Batteries were constructed for the four groups of samples, and their charge and discharge capacities were measured at a current density of 0.5 mA g⁻¹

**Table 2:** Conductivity and Deposition of Four PANI/CC Samples

Sample	1	2	3	4
Conductivity (S·cm^−1^)	0.58	1.52	1.58	1.61
Deposition (mg·cm^−2^)	0.25	0.73	1.35	2

**Fig. 10:**
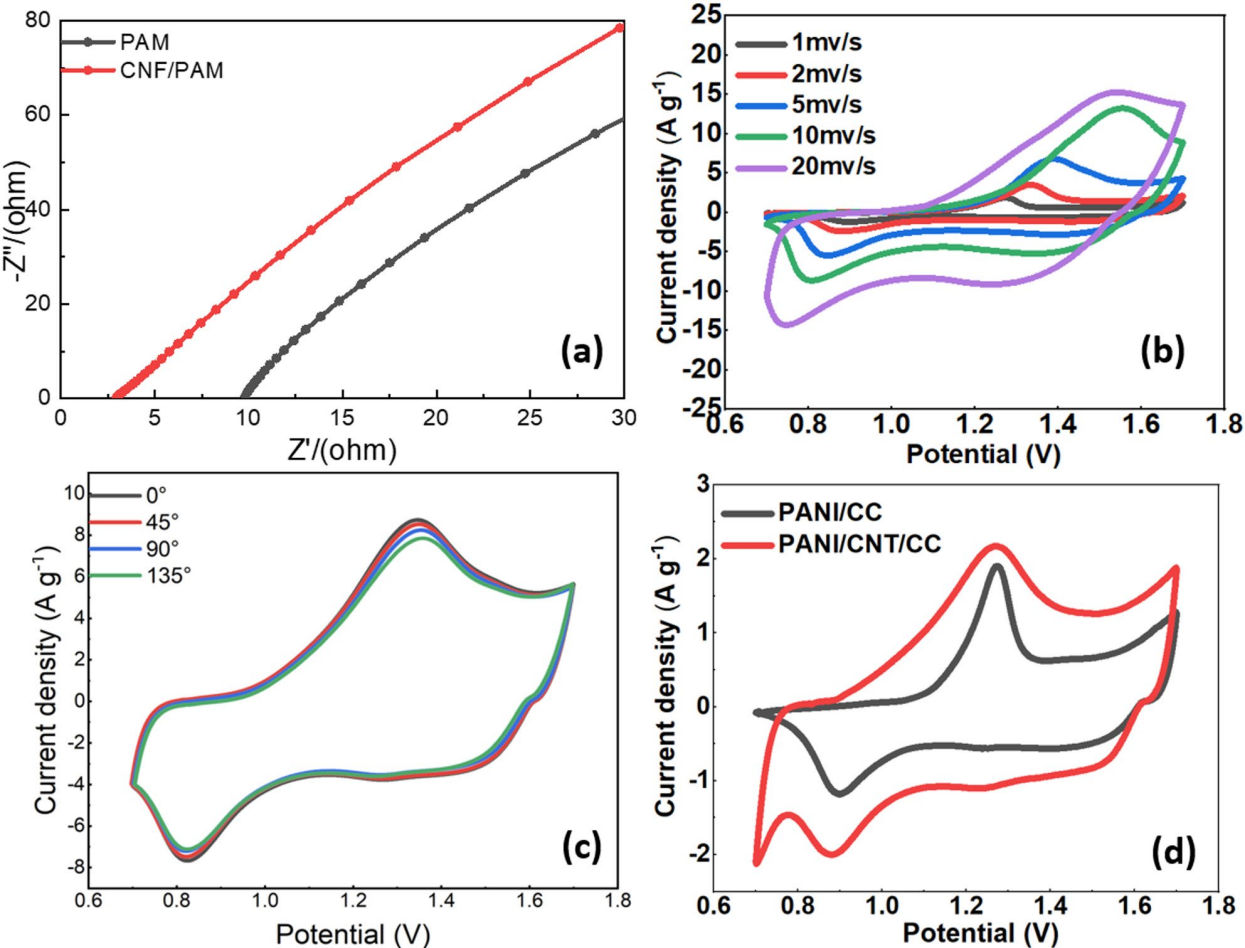
AC impedance spectrum of PAM and CNF/PAM gel electrolyte (**a**) CV curves of flexible PANI/CNT/CC | CNF/PAM | Zn batteries at different scan rates (**b**). CV curves of flexible PANI/CNT/CC | CNF/PAM | Zn batteries bent at different angles (**c**) and comparison of CV curves of two types of batteries at 1 mV/s (**d**)

Figure 10a shows the Nyquist plots of PAM and CNF/PAM electrodes, complementing their electrochemical kinetics. A high-frequency intercept on real axis refers to the solution resistance (R_s_), whereas the semicircle diameter signifies the charge transfer resistance (R_ct_). PAM exhibited a smaller semicircle than CNF/PAM, demonstrating a lower R_ct_ and better electron transfer due to unhindered electron transport. In low-frequency region, both plots showed an inclined line, i.e., a characteristic of Warburg impedance, related to ion diffusion. The steeper slope of CNF/PAM suggests higher ion diffusion resistance, which arising from increased structural density and reduced conductive pathways because of CNF incorporation.

Besides that, the wettability of hydrogels affects their ionic conductivity. Water absorption rate of the CNF/PAM double network gel could reach 1265%, i.e., a 55% improvement to the absorption rate of the PAM gel. CNF network introduces a considerable number of hydrophilic groups, which enhances the water uptake of PAM gel thereby reducing the R_s_ of CNF/PAM as showed in Fig. 10a. The ionic conductivity and water retention of CNF/PAM hydrogel are greatly improved than PAM-only gels due to the CNF network’s hydrophilicity. As a result, the CNF/PAM hydrogel exhibited higher ionic conductivity (2.3 × 10⁻² S cm⁻²) compared to PAM, consistent with its lower measured Rs in the Nyquist analysis.

Figure 10b represents the voltammetric profile of flexible PANI/CNT/CC|CNF/PAM|Zn battery within the voltage window of 0.7 to 1.7 V at scan rates of 1 mV s^−1^, 2 mV s^−1^, 5 mV s^−1^, 10 mV s^−1^, and 20 mV s^−1^. Two redox peaks are evident on the CV curve, namely an oxidation peak near 1.3 V and a reduction peak near 0.9 V. As the scan rate increases, the area enclosed by the CV curve also increases. Figure 10c shows the CV curves of the flexible PANI/CNT/CC|CNF/PAM|Zn battery at different bending angles in the voltage window of 0.7–1.7 V and at a scan rate of 2 mV s^−1^. The consistent CV response at different bending angles confirms excellent mechanical flexibility and electrochemical integrity. Compared with the poor bending stability of batteries directly using PANI/CC and PAM gel electrolytes, the CV curve of the flexible PANI/CNT/CC|CNF/PAM|Zn battery exhibits minimal variation when subjected to bending, thereby demonstrating its exceptional bending stability. The notable enhancement in performance can be attributed to two key factors. Firstly, the porous structure of the current collector is effective in preventing the positive electrode active material from falling off under bending conditions. Secondly, the CNF/PAM double network gel electrolyte, which exhibits excellent physical and electrochemical properties, is capable of significantly improving the issue of insufficient contact between the electrolyte and the electrode.

Figure 10d presents a comparison of the CV curves of the two batteries within the specified voltage window of 0.7 to 1.7 V and at a fixed scan rate of 1 mV s⁻¹. It can be observed that the area enclosed by the CV curve of the flexible PANI/CNT/CC positive electrode is larger, which indicates that its specific capacity has been markedly enhanced. Furthermore, the reduction in overpotential can be attributed to the three-dimensional conductive network constructed by the composite current collector, which has the effect of significantly improving the conductivity of the electrode material. The conclusions demonstrate that the composite cathode and the double network gel electrolyte can markedly enhance the specific capacity of the flexible positive electrode and improve the bending stability of the battery.

**Fig. 11:**
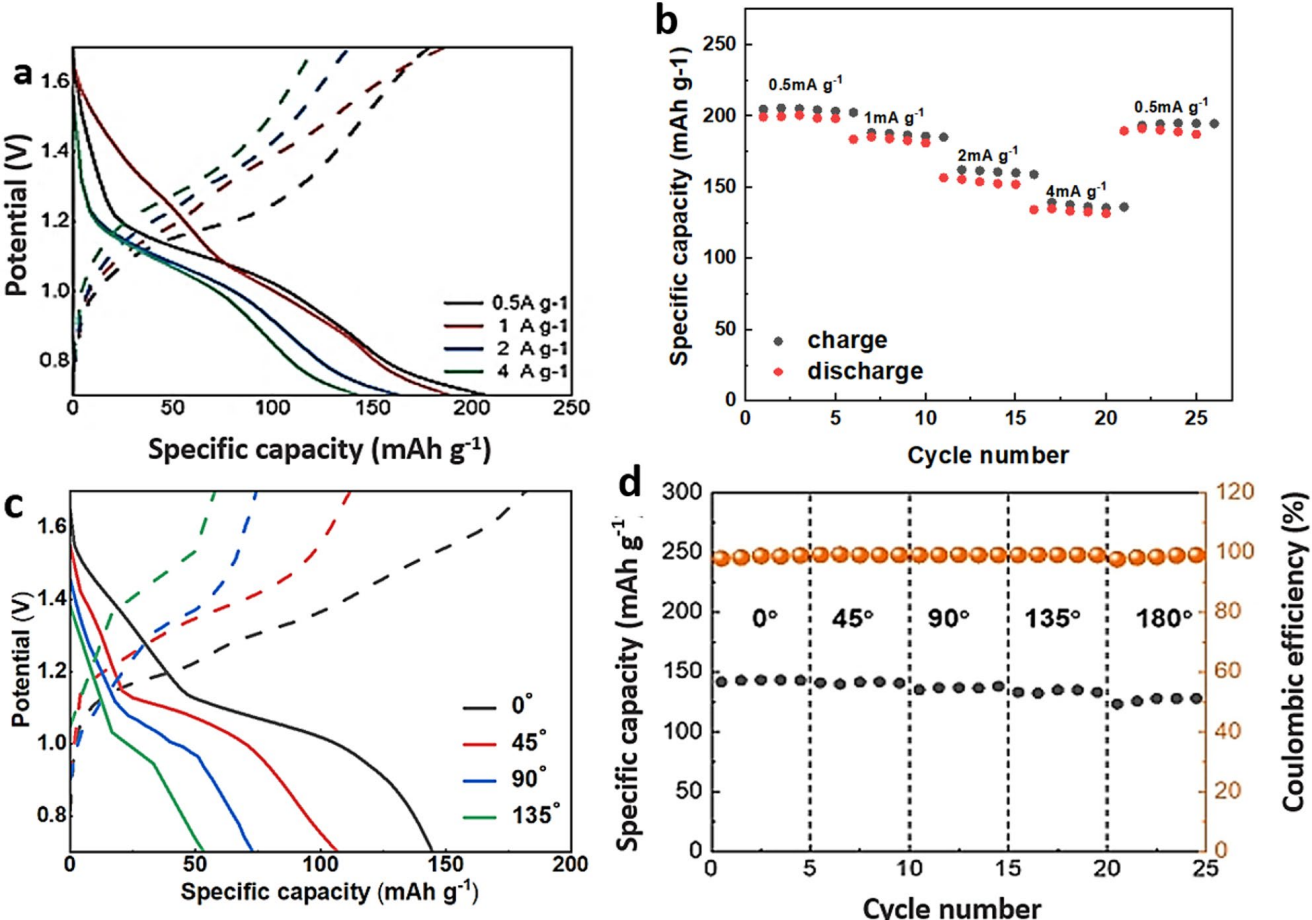
Specific capacity-voltage curve of flexible PANI/CNT/CC|CNF/PAM|Zn battery at varied current densities (**a**) multiplication performance (**b**) specific capacity-voltage curves of PANI/CNT/CC|CNF/PAM|Zn battery at different angles (**c**) cyclic stability in bending state (**d**)

To further evaluate the performance of flexible PANI/CNT/CC|CNF/PAM|Zn batteries. The specific capacity was measured at current densities of 0.5 A g^−1^, 1 A g^−1^, 2 A g^−1^, and 4 A g^−1^. As shown in Fig. 11a and b, the specific capacity and rate performance of flexible batteries at various current densities have been greatly improved. The initial lower charge capacity is attributed to minor irreversible side reactions, which is a common feature in the initial cycling of PANI based cathodes. At a current density of 0.5 A g^−1^, the discharge specific capacity is increased to 210 mA h g^−1^, representing a 45% enhancement in comparison to the previous measurement. Upon increasing the current density to 1 A g^−1^, the discharge specific capacity is 185.4 mA h g^−1^, indicating a 78% increase. An increase to 2 A g^−1^ results in a specific capacity of 164.8 mA h g^−1^, representing a 126.6% enhancement. At a high current density of 4 A g^−1^, the capacity can still reach 139.6 mA h g^−1^, which is 66% of the specific capacity at a current density of 0.5 A g^−1^. In comparison with PANI/CC-zinc ion battery, the specific capacity has increased by 179%. This further substantiates that the utilization of PANI/CNT/CC flexible electrodes and CNF/PAM double network gel electrolyte can not only enhance the specific capacity of the battery but also facilitate the battery’s attainment of superior rate performance. It is evident that an increase in the charge and discharge current results in a more pronounced enhancement in rate performance. Figure 11c shows the variation in specific capacity with varied bending angles i.e., 0°, 45°, 90° and 135°. The voltage-time output curves exhibit variation in amplitude and shape across different bending angles. It can be attributed not to changes in the intrinsic battery performance, but rather to external mechanical factors such as contact resistance fluctuation, internal strain redistribution, misalignment or compression. Minor changes in physical configuration can impact the measured voltage, even when the underlying electrochemical behavior remains unchanged.

The battery demonstrated satisfactory cycle stability at varying bending angles. Subsequently, it was ascertained in Fig. 11d that when the device was subjected to a bending angle of 0° to 45°, 90°, 135° and 180°, respectively, and at a current density of 2 A g^−1^, the battery demonstrated a specific capacity of 145, 142, 137, 135 and 127 mA hg^−1^. Under varying bending angles (0° to 180°), the device maintained high capacity (127–145 mAh g⁻¹) with minimal coulombic efficiency drop, except at 180°, likely due to temporary loss of interfacial contact due to inadequate contact between the battery and the gel electrolyte (Fig. 11d. This confirms that the dual-network gel maintains structural integrity and adhesion under stress. This outcome can be attributed to the combined influence of the composite cathode and the dual-network gel electrolyte. Composite cathode is capable of not only significantly increasing the specific surface area but also adapting to the volume change of PANI during the charging and discharging process. Furthermore, it can prevent the shedding of the cathode active material during the bending process, thereby significantly improving the specific capacity and bending stability of the battery. Three-dimensional positive electrode current collector network with enhanced conductivity and the gel electrolyte with augmented ion mobility diminish the interface impedance, enhance the electrode-electrolyte contact, and inhibit the growth of zinc dendrites. This results in a reduction in internal resistance during battery operation and a notable enhancement in the battery’s performance.

**Fig. 12:**
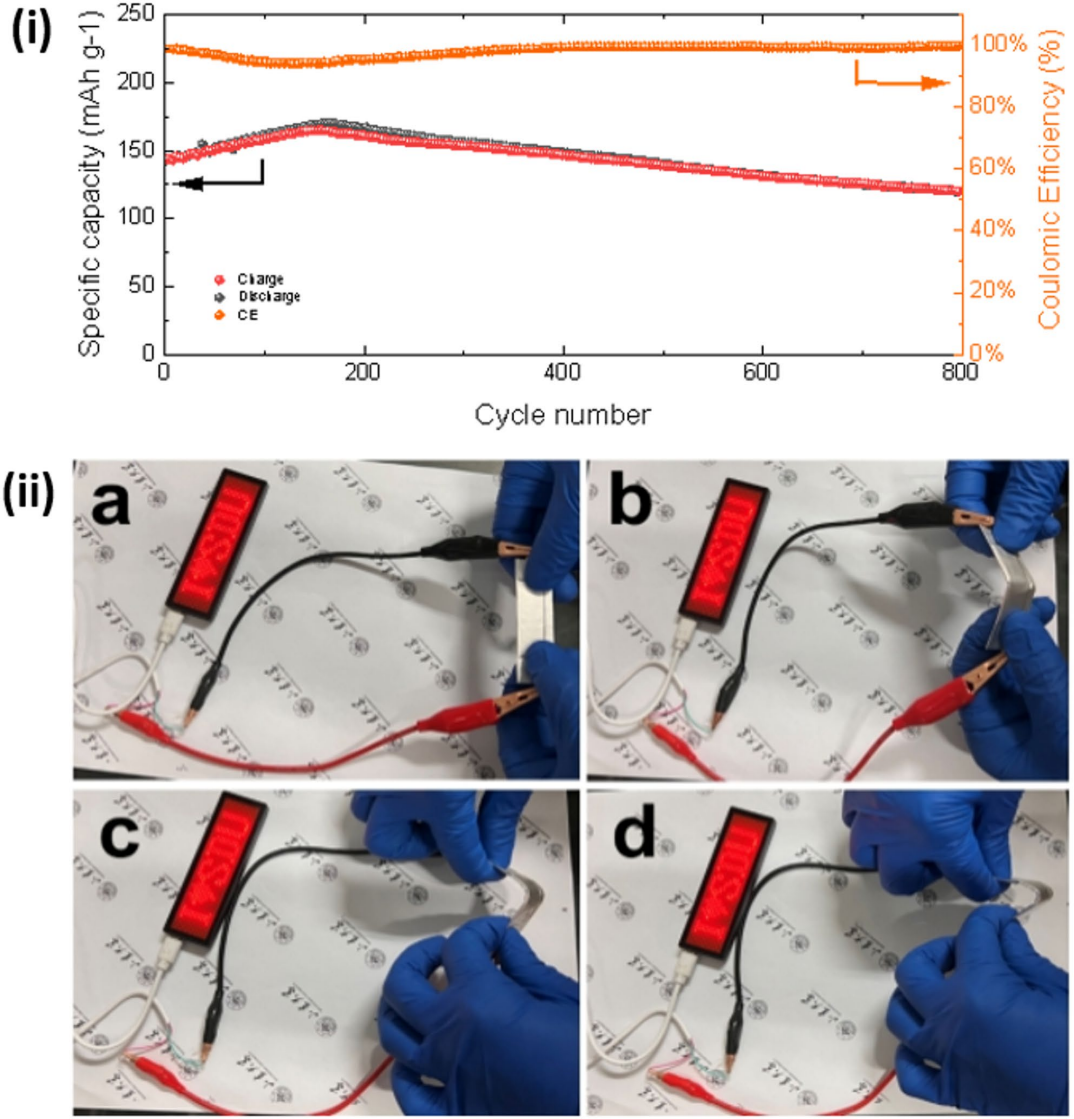
Performance test of flexible PANI/CNT/CC|CNF/PAM|Zn battery charging and discharging cycles (i). Flexible PANI/CNT/CC|CNF/PAM|Zn battery for LED power supply display (ii)

Figure 12i displays the charge and discharge cycle performance of the flexible PANI/CNT/CC|CNF/PAM|Zn battery at a current density of 2 A g^−1^. It can be observed that the charge and discharge capacity initially increase and subsequently declines with the increase in the number of cycles. Since the oxidation process of PANI during the initial cycle contributes supplementary capacity to the battery, the specific charge and discharge capacity continues to decline. In the initial cycle, the battery exhibits a discharge specific capacity of 143.6 mA hg^−1^. Following 800 cycles, the reversible capacity remains at 120.1 mA hg^−1^, with a capacity retention rate of approximately 83.6% and an average coulombic efficiency of 98.7%. The battery exhibits excellent cycle stability. In comparison to the battery prior to optimization, the values for coulombic efficiency and reversible capacity have been significantly enhanced.

A battery is assembled to power the LED light as shown in Fig. 12ii. The battery is bent at 0°, 45°, 90°, and 135°. During the whole process, the LED light can always display normally without extinguishing or flickering. This shows that battery has good flexibility and can meet the power supply needs of wearable devices. It has great application prospects in the field of flexible energy storage in the future. The electrochemical performance of the PANI/CNT/CC|CNF/PAM|Zn battery originates from synergistic interaction between the three-dimensional cathode architecture and the dual-network hydrogel electrolyte. CNT/CC forms a conductive porous framework that increases the electrochemically active surface area and facilitates uniform deposition of PANI, thereby enhancing electron transport during redox reactions. Meanwhile, the CNF/PAM hydrogel electrolyte provides interconnected ion transport channels with high water retention and ionic conductivity. Hydroxyl and carboxyl functional groups present in CNFs interact with Zn^2+^ ions, promoting uniform ion distribution and suppressing dendrite formation at the electrode interface. Furthermore, the porous PANI/CNT/CC structure allows deeper penetration of the hydrogel electrolyte, resulting in intimate electrode-electrolyte contact and reduced charge-transfer resistance. Consequently, the integrated electrode-electrolyte configuration establishes an efficient electron-ion transport pathway, leading to improved specific capacity, rate capability, and cycling stability. Table 3 gives a brief comparison of our battery composite with recently reported similar materials.

**Table 3:** Comparison of PANI/CNT/CC|CNF/PAM|Zn with similar materials for ZIBs

Cathode material	Electrolyte	Specific capacity (mA h/g)	Cycle stability (No. of cycles)	Capacity retention (%)	References
PANI@CNTs//Zn	PGE	155 at 0.2 A/g	1500	99	[49]
PANI@CF	PVA	215 at 0.2 A/g	2500	88	[50]
PANI@V_2_O_5_	2 M Zn(OTf)_2_ in PEG	120 at 0.2 A/g	200	65	[51]
MnOx/PPy/ITO/PET	Gelatin/borax/1MZnSO4/ CdTe QDs	110 at 0.2 A/g	200	–	[52]
MnO2/CNT	PAM/ 2 M ZnSO4/ 0.1 M MnSO4	60 at 0.1 A/g	500	98	[53]
PANI/CNT/CC	CNF/PAM Hydrogel	210 at 0.5 A/g	1000	91	This work

## Conclusion

This work demonstrates a system-level integration of a 3D conductive cathode with a dual-network hydrogel electrolyte that can simultaneously optimize electron transport, ion diffusion, and interfacial stability in flexible zinc-ion batteries. Herein, the optimal preparation conditions for a polyaniline cathode were investigated and optimized as a composite cathode and gel electrolyte for ZIB. Initially, a three-dimensional CNT/CC current collector was constructed, and a PANI/CNT/CC flexible cathode was prepared. The PAM gel was modified with CNF to obtain a double-grid structure CNF/PAM gel. The optimized flexible battery exhibits a high specific capacity of 210 mA hg^− 1^ at a current density of 0.5 A g^− 1^, representing a 45% enhancement compared to the pre-optimized configuration. At a high current density of 4 A g^− 1^, the capacity remains at 139.6 mA hg^− 1^, representing a 179% improvement over the previous optimization. Following 800 charge and discharge cycles at a current density of 2 A g^− 1^, the battery exhibited a capacity retention rate of approximately 83.6%, with an average coulombic efficiency of 98.7%. The results achieved are of an excellent standard. Even at a large bend angle of 135°, the battery can deliver normal power. The battery can meet the current demand for flexible wearable batteries.

## Data Availability

All the data is confined in the manuscript, and no supplementary files are available. Data may be made available on reasonable request from the corresponding authors.

## Abbreviations

ZIB: Zinc ion battery
CNT: Carbon nanotube
PANI: Polyaniline
CC: Carbon cloth
CNF: Carbon nanofibers
PAM: Polyacrylamide
GPE: Gel polymer electrolyte
CVD: chemical vapor deposition
CV: Cyclic voltammetry
GCD: Galvanic charge/discharge
EIS: Electrochemical impedance spectroscopy
LSV: Linear sweep voltammetry
SEM: Scanning electron microscopy
TEM: Transmission electron microscopy

## References

[1] Sun B, et al. Activating Gel Polymer Electrolyte Based Zinc-Ion Conduction with Filler-Integration for Advanced Zinc Batteries. ACS Appl Materials Interfaces. 2023;15(31):37916–24. 10.1021/acsami.3c06702 37491187

[2] Zamarayeva AM, et al. Flexible and stretchable power sources for wearable electronics. Sci Adv. 2017;3(6):e1602051. 28630897 10.1126/sciadv.1602051 PMC5473674

[3] Wang Z, et al. Accidents involving lithium-ion batteries in non-application stages: incident characteristics, environmental impacts, and response strategies. BMC Chem. 2025;19(1):94. 40223134 10.1186/s13065-025-01445-x PMC11995524

[4] Yao K, et al. Designing sodium alloys for dendrite-free sodium‐metal batteries. Information Functional Materials. 2024;1(2):242–63.

[5] Zhang K, et al. Design and fabrication of wearable electronic textiles using twisted fiber-based threads. Nat Protoc. 2024;19(5):1557–89. 38429518 10.1038/s41596-024-00956-6

[6] Xu Z, et al. Smart Textiles for Personalized Sports and Healthcare. Nano-Micro Lett. 2025;17(1):1–39. 10.1007/s40820-025-01749-6 PMC12031719 40278986

[7] Hu J, Dong M. Recent advances in two-dimensional nanomaterials for sustainable wearable electronic devices. J Nanobiotechnology. 2024;22(1):63. 38360734 10.1186/s12951-023-02274-7 PMC10870598

[8] Liu A, et al. Reviewing metal fluorides as the cathode materials for high performance Li batteries. Information Functional Materials. 2024;1(1):26–67.

[9] Chao D, et al. Roadmap for advanced aqueous batteries: from design of materials to applications. Sci Adv. 2020;6(21):eaba4098. 32494749 10.1126/sciadv.aba4098 PMC7244306

[10] Joshi B, et al. Progress and potential of electrospinning-derived substrate-free and binder-free lithium-ion battery electrodes. Chem Eng J. 2022;430:132876.

[11] Tang B, et al. Issues and opportunities facing aqueous zinc-ion batteries. Energy Environmental Science. 2019;12(11):3288–304.

[12] Wu TH, et al. Nanoscale design of zinc anodes for high-energy aqueous rechargeable batteries. Mater Today Nano. 2019;6:100032.

[13] Lu Y, et al. Rational design and demonstration of a high-performance flexible Zn/V2O5 battery with thin-film electrodes and para-polybenzimidazole electrolyte membrane. Energy Storage Mater. 2020;27:418–25.

[14] McCoy DE, et al. Structural absorption by barbule microstructures of super black bird of paradise feathers. Nat Commun. 2018;9(1):1. 29317637 10.1038/s41467-017-02088-w PMC5760687

[15] Liu J, et al. A flexible alkaline rechargeable Ni/Fe battery based on graphene foam/carbon nanotubes hybrid film. Nano Lett. 2014;14(12):7180–7. 25402965 10.1021/nl503852m

[16] Wang H, et al. An ultrafast nickel–iron battery from strongly coupled inorganic nanoparticle/nanocarbon hybrid materials. Nat Commun. 2012;3(1):917. 22735445 10.1038/ncomms1921

[17] Liu J, et al. A flexible quasi-solid-state nickel–zinc battery with high energy and power densities based on 3D electrode design. Adv Mater. 2016;28(39):8732–9. 27562134 10.1002/adma.201603038

[18] Huang X, et al. High-Performance Electrospun Poly(vinylidene fluoride)/Poly(propylene carbonate) Gel Polymer Electrolyte for Lithium-Ion Batteries. J Phys Chem C. 2015;119(50):27882–91.

[19] Anshari R, et al. Raman and ATR-FTIR unmask crystallinity changes and carboxylate group and vinyl group accumulation in natural weathering polypropylene microplastics. Sci Rep. 2025;15(1):2518. 39833276 10.1038/s41598-025-85837-y PMC11747500

[20] Ghaffari F, Shekaari H. Application of fatty acid-based eutectic mixture as a phase change material in microencapsulation of drugs: preparation, characterization and release behavior. BMC Chem. 2025;19(1):1–14. 39962482 10.1186/s13065-025-01406-4 PMC11834564

[21] Verma S, et al. A facile synthesis of novel polyaniline/graphene nanocomposite thin films for enzyme-free electrochemical sensing of hydrogen peroxide. Mol Syst Des Eng. 2022;7(2):158–70.

[22] Peng H, et al. A coral-like polyaniline/barium titanate nanocomposite electrode with double electric polarization for electrochromic energy storage applications. J Mater Chem A. 2021;9(3):1669–77.

[23] Kong L-B, et al. MWNTs/PANI composite materials prepared by in-situ chemical oxidative polymerization for supercapacitor electrode. J Mater Sci. 2008;43(10):3664–9.

[24] Yan J, et al. Preparation of a graphene nanosheet/polyaniline composite with high specific capacitance. Carbon. 2010;48(2):487–93.

[25] Li W, et al. Strong and robust polyaniline-based supramolecular hydrogels for flexible supercapacitors. Angew Chem Int Ed Engl. 2016;55(32):9196–201. 27328742 10.1002/anie.201603417

[26] Mahmood A, Muhmood T, Ahmad F. Carbon nanotubes heterojunction with graphene like carbon nitride for the enhancement of electrochemical and photocatalytic activity. Mater Chem Phys. 2022;278:125640.

[27] Jadhav VV, et al. Annealing environment effects on the electrochemical behavior of supercapacitors using Ni foam current collectors. Mater Res Express. 2018;5(12):125004.

[28] Lu L, et al. Template-free synthesis of nanoporous nickel and alloys as binder-free current collectors of Li ion batteries. ACS Appl Nano Mater. 2018;1(5):2206–18. 29911687 10.1021/acsanm.8b00284 PMC5999232

[29] Mi H, et al. Three-dimensional network structure of silicon-graphene-polyaniline composites as high performance anodes for Lithium-ion batteries. Electrochim Acta. 2016;190:1032–40.

[30] Shen J, et al. High-Performance Asymmetric Supercapacitor Based on Nanoarchitectured Polyaniline/Graphene/Carbon Nanotube and Activated Graphene Electrodes. ACS Appl Materials Interfaces. 2013;5(17):8467–76. 10.1021/am4028235 23931572

[31] Cong H-P, et al. Flexible graphene–polyaniline composite paper for high-performance supercapacitor. Energy Environ Sci. 2013;6(4):1185–91.

[32] He H, et al. Fabrication of 3D ordered honeycomb-like nitrogen-doped carbon/PANI composite for high-performance supercapacitors. Appl Surf Sci. 2019;484:1288–96.

[33] Shirmardi A, et al. Enhanced photocatalytic performance of ZnSe/PANI nanocomposites for degradation of organic and inorganic pollutants. Appl Surf Sci. 2018;462:730–8.

[34] Shalini V, et al. Design and fabrication of PANI/GO nanocomposite for enhanced room-temperature thermoelectric application. Appl Surf Sci. 2019;493:1350–60.

[35] Yang H, et al. Covalent functionalization of chemically converted graphene sheets via silane and its reinforcement. J Mater Chem. 2009;19(26):4632–8.

[36] Liu X, et al. Highly compressible three-dimensional graphene hydrogel for foldable all-solid-state supercapacitor. J Power Sources. 2018;384:214–22.

[37] Zou Y, et al. Hydrothermal direct synthesis of polyaniline, graphene/polyaniline and N-doped graphene/polyaniline hydrogels for high performance flexible supercapacitors. J Mater Chem A. 2018;6(19):9245–56.

[38] Stankovich S, et al. Synthesis of graphene-based nanosheets via chemical reduction of exfoliated graphite oxide. Carbon. 2007;45(7):1558–65.

[39] Hualan W, et al. Effect of graphene oxide on the properties of its composite with polyaniline. ACS Appl materials interfaces. 2010;2(3):821–8. 10.1021/am900815k 20356287

[40] Fan X, et al. Deoxygenation of exfoliated graphite oxide under alkaline conditions: A green route to graphene preparation. Adv Mater. 2008;20(23):4490–3.

[41] P Singh R, Pal S, Ali AJAML. Novel biodegradable polymeric flocculants based on cationic polysaccharides. Adv Mater Lett. 2014;5(1):24–30.

[42] Malik S, et al. Ultrasound-assisted surface modification of cellulose isolated from rice husk to impart hydrophobicity. J Pharm Innov. 2023;12:2601–5.

[43] Biswal DR, Singh RP. Characterisation of carboxymethyl cellulose and polyacrylamide graft copolymer. Carbohydr Polym. 2004;57(4):379–87.

[44] Chang C, Duan B, Zhang LJP. Fabrication and characterization of novel macroporous cellulose–alginate hydrogels. Polymer. 2009;50(23):5467–73.

[45] Hongfei L, et al. Waterproof and Tailorable Elastic Rechargeable Yarn Zinc Ion Batteries by a Cross-Linked Polyacrylamide Electrolyte. ACS Nano. 2018;12(4):3140–8. 29589438 10.1021/acsnano.7b09003

[46] Wang D, et al. A nanofibrillated cellulose/polyacrylamide electrolyte-based flexible and sewable high‐performance Zn–MnO_2_ battery with superior shear resistance. Small Weinheim Bergstr. 2018;14(51):1803978. 10.1002/smll.201803978 30444576

[47] Biswal D, Singh R. Flocculation studies based on water-soluble polymers of grafted carboxymethyl cellulose and polyacrylamide. J Appl Polym Sci. 2006;102(2):1000–7.

[48] Li Q, et al. Self-healable hydrogel electrolyte toward high-performance and reliable quasi-solid-state Zn–MnO_2_ batteries. ACS Appl Mater Interfaces. 2019;11(42):38762–70. 31583879 10.1021/acsami.9b13553

[49] Abbasi R, et al. Low-temperature direct ammonia fuel cells: Recent developments and remaining challenges. Curr Opin Electrochem. 2020;21:335–44.

[50] Li D, et al. High-performance compressible zinc ion battery based on melamine foam‐derived electrodes. Small Struct. 2022;3(8):2200027.

[51] Ciurduc DE, et al. Molecular crowding bi-salt electrolyte for aqueous zinc hybrid batteries. Energy Storage Mater. 2022;53:532–43.

[52] Zhu M, et al. Light-permeable, photoluminescent microbatteries embedded in the color filter of a screen. Energy Environ Sci. 2018;11(9):2414–22.

[53] Ma L, et al. Initiating a mild aqueous electrolyte Co_3_O_4_/Zn battery with 2.2 V-high voltage and 5000-cycle lifespan by a Co (iii) rich-electrode. Energy Environ Sci. 2018;11(9):2521–30.

